# Shear Bond Strength Between Artificial Teeth and Denture Base Resins Fabricated by Conventional, Milled, and 3D-Printed Workflows: An In Vitro Study

**DOI:** 10.3390/ma18194590

**Published:** 2025-10-03

**Authors:** Giulia Verniani, Fatemeh Namdar, Ovidiu Ionut Saracutu, Alessio Casucci, Marco Ferrari

**Affiliations:** 1 Department of Prosthodontics, University of Siena, 53100 Siena, Italy; f.namdar@student.unisi.it (F.N.); ferrarm@gmail.com (M.F.); 2 Department of Medical Biotechnologies, Division of TMJ and Oral Pain, University of Siena, 53100 Siena, Italy; ovidio.saracutu@gmail.com; 3 Division of Gerodontology and Removable Prosthodontics, University Clinics of Dental Medicine, University of Geneva, 1205 Geneva, Switzerland; alessiocasucci@gmail.com

**Keywords:** complete denture, digital dentistry, 3D printing, digital workflow

## Abstract

Background: The adhesion between artificial teeth and denture bases is crucial for the longevity of complete dentures. This in vitro study evaluated the shear bond strength (SBS) and failure modes between artificial teeth and denture base resins produced with conventional, milled, and 3D-printed techniques. Materials: A total of 105 specimens were fabricated and assigned to 7 groups (n = 15) combining conventional, milled, or printed denture bases with conventional, milled, or printed teeth. SBS was tested using a universal testing machine, and failure modes were classified as adhesive, cohesive, or mixed. Data were analyzed with one-way ANOVA and Tukey’s post hoc test (α = 0.05). Results: SBS significantly varied among groups (*p* < 0.001). The conventional base–conventional tooth group (CB-CT) showed the highest bond strength (14.9 ± 3.69 MPa), while the printed base–milled tooth group (PB-MT) had the lowest (6.58 ± 3.41 MPa). Milled base groups showed intermediate values (11.7–12.4 MPa). Conclusions: Bond strength between denture teeth and denture bases depends on the fabrication workflow. Conventional heat-cured PMMA bases exhibited the most reliable adhesion, while milled bases demonstrated satisfactory performance with optimized bonding. Printed bases showed reduced and variable adhesion, suggesting the need for improved bonding protocols before their widespread clinical application in definitive prostheses.

## 1. Introduction

Complete dentures remain a widely used treatment option for fully edentulous patients, although their conventional fabrication is labor-intensive, requiring multiple clinical visits and significant laboratory work. Traditionally, these dentures are produced via the lost-wax technique using heat-polymerized polymethyl methacrylate (PMMA) bases combined with prefabricated resin teeth [1]. It has been reported that 22% to 30% of traditional denture repairs, particularly in the anterior area, are caused by debonding of the teeth from the denture base [2]. A secure attachment between denture teeth and the base resin is achieved through both mechanical interlocking and chemical bonding. Various types of denture teeth can be utilized in conventional complete dentures, including standard acrylic resin teeth and those made from partially or highly cross-linked resins. [3]. While this approach has provided dependable outcomes for many years, it involves a time-consuming process that necessitates several clinical interventions and considerable laboratory effort [4,5]. Recently Digital complete denture (DDs) can be produced through subtractive (milling) or (additive manufacturing) 3D printing. DDs provide several benefits, including improved fit accuracy, enhanced mechanical performance, and superior reproducibility, along with reducing chair-time and number of appointments [6]. Among the available digital solutions, milled complete dentures are commonly selected due to their strength and accuracy [7,8]. 3D printing builds objects layer by layer by curing a photosensitive liquid resin offering several advantages over milling, such as reduced material waste, the ability to fabricate multiple dentures at once, and greater ability in reproducing undercuts [9,10]. 3D-printed complete dentures are gaining attention as more affordable alternatives, though their long-term clinical outcomes remain under investigation [11]. The commercial teeth and the resin base have distinct compositions because of the different processing techniques resulting in different shear bond strength values depending on the different processing methods [12]. Bi-layered CAD/CAM discs, such as Ivotion (Ivoclar Vivadent), integrate both base resin and teeth in one monolithic block. These eliminate separate bonding steps entirely and provide a consistent, durable tooth–base interface—with studies showing bond strengths on par with traditional prostheses [13]. Although in some cases due to teeth mounting and arch shape of the patients, the complete denture can’t be produced from a bylayered disk and usually the fabrication process of a digital complete denture begins with two separate stl file—one for the base and one for the teeth. The denture teeth are then joined to the base using different resins depending on the manufacturer. Although various manufacturing approaches can be applied to fabricate the teeth and base, there is limited evidence identifying which combinations yield the most robust bond and structural integrity at the tooth–base interface. Several comparative studies have quantified the shear bond strength (SBS) between denture teeth and denture base resins fabricated through different workflows. Han et al. (2020) reported SBS values ranging from 16.90 MPa to 21.80 MPa for CAD/CAM denture base resins bonded with resin cement, suggesting that digital workflows can approach the strength of conventional methods when optimal bonding protocols are used [14]. Prpić et al. (2020) found that SBS values varied between 12.56 MPa and 18.10 MPa depending on the combination of base and tooth materials, with conventional PMMA-based combinations generally yielding higher bond strengths than digital ones [15]. Similarly, Helal et al. (2022) highlighted the role of surface treatment and observed SBS values ranging from 7.92 MPa to 19.31 MPa, with untreated printed surfaces showing lower adhesion [16]. Mine et al. (2019) conducted a systematic review of 32 studies and reported that shear bond strength (SBS) values for CAD/CAM resin bases ranged from 12.56 MPa to 18.10 MPa, depending on surface pretreatment and bonding protocols [17]. These values were generally lower than those achieved with conventional heat-polymerized PMMA bases, which often showed SBS values exceeding 20 MPa under comparable testing conditions. Limited information is available on the shear bond strength (SBS) between denture teeth and denture base resins fabricated using a 3D printing. The purpose of this in vitro study was to evaluate the adhesion between artificial teeth and resin bases using conventional and digital fabrication methods. The null hypothesis was that the adhesion between teeth and denture bases would not differ in the different workflows.

## 2. Materials and Methods

A total of 105 specimens were prepared and allocated to 7 groups (n = 15/group) combining base type (conventional, milled, printed) and tooth type (conventional, milled, printed as reported in Table 1):

CB-CT: conventional base (ProBase Hot; Ivoclar Vivadent), conventional tooth (SPE; Ivoclar Vivadent).

MB-CT: milled base (pre-polymerized PMMA), conventional tooth (SPE).

MB-PT: milled base, printed tooth (Varseo Smile Teeth; BEGO).

MB-MT: milled base (Ivotion Base; Ivoclar Vivadent), milled tooth (Ivotion Dent Multi; Ivoclar Vivadent).

PB-CT: printed base (V-Print; VOCO), conventional tooth (SPE).

PB-PT: printed base, printed tooth (Varseo Smile Teeth; BEGO).

PB-MT: printed base, milled tooth (Ivotion Dent Multi).

Sample size was calculated to achieve 88% statistical power at an alpha level of 0.05, based on estimates from previous studies.

Cylindrical base disks (Ø 5.0 mm × 2.5 mm height) were used to standardize the bonded area and specimen height across groups, as reported in previous studies [15,16]. The bonded interface corresponded to the circular face (Ø 5.0 mm) of the base disk (area ≈ 19.6 mm^2^).

### 2.1. Conventional Group

The CB-CT group, consisting of conventional denture base resin and artificial teeth, served as the control group. Base disks (5 mm diameter × 2.5 mm height) were digitally designed with CAD software (Meshmixer 3.5, Autodesk) and fabricated by milling wax (Star wax blank Dentaurum, Bologna, Italy) to standardize the samples. Wax disks were bonded to the cervical surface of conventional upper molars tooth number 16 (SPE, Ivoclar Vivadent, Liechtenstein) and invested in type IV dental gypsum. The lost-wax technique was used for processing, following the manufacturer’s instructions. The resin (Probase Hot, Ivoclar Vivadent, Liechtenstein) was packed and polymerized under 200 bars of pressure at 70 °C for 30 min, followed by boiling at 100 °C for an additional 30 min. After deflasking and cleaning, the specimens were finished and polished.

### 2.2. Digital Groups

**Figure 1:**
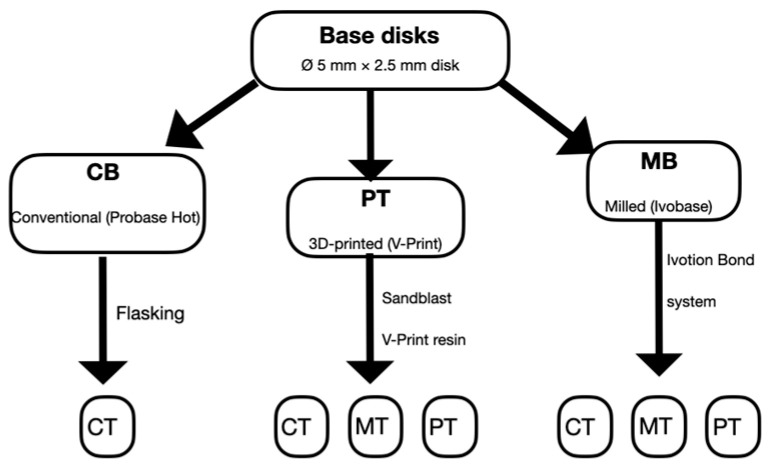
Flowchart of the assembly procedure of all the samples. CB: conventional base, MB: milled base, PT: printed base, CT: conventional tooth, PT: printed tooth, MT: milled tooth.

The same maxillary molar (tooth 16, SPE, Ivoclar Vivadent, Liechtenstein) was digitally scanned (Trios 5, 3Shape) to standardize tooth geometry across groups. STL files were used to fabricate artificial teeth either by milling (Ivotion Dent Multi, Ivoclar Vivadent, Liechtenstein) using a 5-axis milling unit (PM7) or by 3D printing (Varseo Smile Teeth, Bego) using a DLP printer (Asiga MAX UV, 385 nm, 62 μm resolution, Asiga, Sydney, Australia). Then, Liquidtech BT was used to clean the samples for 20 min using the BB Wash machine (Meccatronicore S.R.L., Pergine Valsugana, TN, Italy). All the samples were polymerized for 20 min with a BB cure machine (Model MTC-BB-CURE-COMPACT, Meccatronicore S.R.L.). Base disks, from the same stl file of the conventional group (5 mm diameter × 2.5 mm height), were fabricated by either milling from Ivotion Dent Multi (MB groups) or printing with V-Print resin (PB groups, VOCO GmbH, Cuxhaven, Germany). All printed components were post-processed according to manufacturer protocols. For bonding, MB disks were bonded to CT, MT, and PT teeth using the Ivotion Bond system (Ivoclar Vivadent, Liechtenstein). PB disks and respective teeth were sandblasted (50 μm Al_2_O_3_, 2 bar, 10 s), bonded under 1 kg pressure using V-Print resin (VOCO), light-cured for 1 min, and subsequently post-polymerized for 15 min (HiLite Power 3D). The assembly procedure of all the samples is summarized in Figure 1.

**Figure 2:**
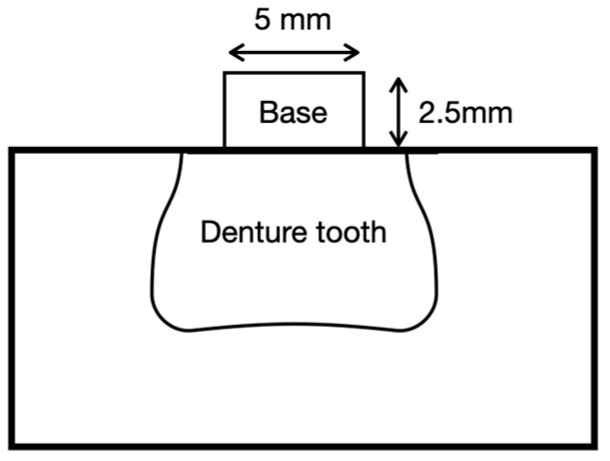
Schematic of denture tooth-denture base resin specimens.

All specimens were embedded in autopolymerizing PMMA resin (Probase Clear, Ivoclar Vivadent) using standardized silicone molds (FlexiForm, Struers, Ballerup, Denmark) to obtain a stable base for testing. Final samples were stored in distilled water at 37 °C for 48 h prior to testing. A schematic of the definitive specimen is shown in Figure 2.

Shear bond strength (SBS) was assessed using a universal testing machine (5567 Universal Testing Machine; Instron Ltd., Norwood, MA USA) at a crosshead speed of 1 mm/min [18]. The maximum load at fracture was recorded in Newtons (N), and SBS was calculated by dividing the load(N) by the bonding area (π × 2.52 mm). 

All fractured specimens were subsequently examined under ×9 magnification using a handheld loupe (Zeiss) to evaluate the nature of failure. Failure modes were categorized as adhesive, cohesive within the tooth or base resin, or mixed.

### 2.3. Statistical Analysis

The assumption of normality was validated by the Kolmogorov–Smirnov test (*p* = 0.200). Effect size analysis indicated a large effect of material group on SBS (η^2^ = 0.418).

Statistical analysis was performed using one-way analysis of variance (ANOVA) to compare SBS values across the experimental groups. Post hoc comparisons were conducted using Tukey’s HSD test, with a significance level set at α = 0.05. The normality of data distribution was verified using the Shapiro–Wilk test.

## 3. Results

The mean SBS values and standard deviations for each group are summarized in Table 2. The CB-CT group, representing the conventional workflow with heat-polymerized PMMA and conventional teeth, exhibited the highest bond strength (14.9 ± 3.69 MPa). In contrast, the PB-MT group, which combined printed base resin and milled teeth, showed the lowest bond strength (6.58 ± 3.41 MPa). Intermediate SBS values were recorded for groups utilizing milled bases, such as MB-CT (12.43 ± 2.31 MPa), MB-PT (12.16 ± 3.14 MPa), and MB-MT (11.71 ± 2.91 MPa), with no statistically significant differences among them (*p* > 0.05). Similarly, the PB-CT and PB-PT groups displayed SBS values of 12.28 ± 2.37 MPa and 8.77 ± 3.52 MPa, respectively. A one-way ANOVA revealed significant differences in SBS among the groups (F = 11.727; *p* < 0.001). Post hoc analysis using the Bonferroni test confirmed that PB-MT had significantly lower bond strength compared to CB-CT, MB-CT, MB-PT, and PB-CT (*p* < 0.001).

Representative failure modes observed in each group are summarized in Table 3. Among the 105 specimens analyzed, adhesive failures were most frequent in PB-PT (n = 6), while lower counts were recorded in PB-MT (n = 3) and CB-CT (n = 2).Cohesive failures were predominant in the MB-CT (n = 12), MB-MT (n = 11), and PB-CT (n = 11) groups. Mixed failures were distributed more evenly, with the highest frequency observed in PB-MT (n = 7) and MB-PT (n = 6). When failure mode was analyzed as a factor influencing SBS, specimens exhibiting adhesive failure demonstrated lower bond strength compared to those with cohesive or mixed failures. One-way ANOVA revealed a statistically significant difference in SBS among the failure mode categories (F(3,101) = 13.506, *p* < 0.001). Post hoc analysis confirmed that adhesive failures were associated with significantly lower SBS values (mean: 7.5 ± 4.4 MPa), while cohesive-base and cohesive-tooth failures corresponded to higher SBS (mean: 12.9 ± 3.2 MPa and 11.56–15.57 MPa, respectively, depending on group) as reported in Table 4.

## 4. Discussion

The present study investigated and compared the shear bond strength (SBS) and failure modes among various combinations of denture base resins and artificial teeth fabricated through conventional, milled, and 3D-printed workflows. The highest mean SBS was observed in the CB-CT group (14.9 ± 3.69 MPa), followed by the MB-CT group (12.43 ± 2.31 MPa) and the PB-CT group (12.28 ± 2.37 MPa), while the lowest SBS was recorded in the PB-MT group (6.58 ± 3.41 MPa). The result of the present study clearly indicate that both the fabrication method of the denture base and the type of artificial tooth had measurable and clinically relevant effects on bond durability, thus the null hypothesis was rejected. Notably, even within digitally fabricated groups, milled bases exhibited higher SBS than printed ones, underlining the importance of manufacturing density, cross-linking, and surface reactivity in clinical performance [14]. The lowest SBS values in the present study were observed in the printed groups, particularly PB-PT (8.77 MPa) and PB-MT (6.58 MPa). These results are below the ranges reported in previous studies up to 18 Mpa [14,15,16,17,18,19,20]. The reduced adhesion recorded in the printed groups is consistent with the findings of Helal et al. [16], who emphasized that untreated printed surfaces show markedly weaker bonding. Mine et al. [17] similarly reported that post-polymerized printed resins are chemically inert and lack unreacted monomeric sites required for copolymerization. This suggests that the inherent surface characteristics of 3D-printed resins may limit tooth–base adhesion, unless enhanced by additional surface treatments or optimized bonding protocols. Ibrahim et al. [21] observed similar SBS values (8–13 MPa) for unreinforced printed materials. Our PB-MT value of 6.58 MPa, slightly below this range, supports the interpretation that sandblasting alone is insufficient for achieving durable bonds in printed systems [22]. Aydin et al. [23], observed a mean SBS of 15.4 MPa for the PB-PT combination, reporting statistically superior SBS values of printed groups compared to conventional ones. However, it should be noted that in their study the conventional CT-CB group showed a mean SBS of only 8.8 MPa, considerably lower than the 14.9 MPa recorded for the CT-CB group in the present study. This discrepancy suggests that the statistically significant difference reported by Aydin et al. in favor of the printed group may have been influenced by the unusually low performance of their conventional control, rather than by a truly superior adhesion of the printed base compared to conventional ones. Therefore, the interpretation of their statistical comparison should be made with caution, as differences in material selection, experimental protocols, or processing conditions may account for the divergent outcomes.

In our study, the milled groups (e.g., MB-CT: 12.43 ± 2.31 MPa; MB-PT: 12.16 ± 3.14 MPa) exhibit values slightly below the range of other studies but still demonstrated reliable bonding, confirming that optimized adhesive systems and precise surface finishing can elevate digital bonding performance. These findings also mirror those of Prpić et al. [15], who reported SBS variability between 12.56 and 18.10 MPa depending on the compatibility of base and tooth materials. Consistent with Prpić’s conclusions, our CB-CT and MB-CT groups—both involving chemically similar PMMA components—outperformed digital combinations. Clinically, this suggests that conventional and milled bases may provide a more predictable long-term retention of denture teeth, whereas printed bases might increase the risk of tooth debonding and need for repairs. A closer inspection of the failure modes further substantiates the mechanical hierarchy. In high-performing groups such as CB-CT and MB-PT, cohesive failures were predominant, indicating that the bond strength between base and tooth exceeded the internal strength of one of the substrates. In the CB-CT group, 10 out of 15 specimens failed cohesively, while only 2 showed adhesive failures. These results confirm that heat-cured PMMA bases facilitate robust interfacial polymer integration, likely due to the high degree of monomer conversion and the favorable chemical affinity between PMMA components [24]. In contrast, the fully digital groups especially PB-MT exhibited markedly different behavior [25]. The PB-MT group not only showed the lowest SBS (6.58 ± 3.41 MPa), but also a higher proportion of adhesive and mixed failures. This failure pattern reflects weak interfacial adhesion, suggesting insufficient chemical bonding or surface activation. Since both the base and the milled tooth in PB-MT were pre-polymerized, the lack of reactive surface monomers may have compromised the bonding potential, even with sandblasting and resin cement application. Statistical analysis of failure modes across all groups reinforces this observation. According to Table 4, cohesive failures were associated with the highest SBS (mean: 12.9 ± 3.2 MPa), whereas adhesive failures had the lowest (mean: 7.5 ± 4.4 MPa). The standard deviations, especially in lower performing groups such as PB-MT and PB-PT, were notably large, indicating variability in adhesion that could compromise clinical predictability. The risk of long-term degradation, fatigue failure, or microleakage increases in prostheses with initially lower bond strengths [24,26]. Therefore, while even printed systems may provide adequate initial retention in low-load scenarios (e.g., interim dentures), their application in definitive, full-load-bearing prostheses should be considered with caution. Future studies should investigate the adhesion between clinical environment, where factors such as humidity, thermal cycling, and dynamic occlusal loading may influence the adhesion between denture teeth and denture base resins.

This study suggests that conventional heat-polymerized PMMA bases, when combined with compatible denture teeth, offer the most favorable bonding characteristics. Milled bases, although slightly lower in SBS, still exhibit consistent performance when bonded with appropriate adhesives. In contrast, printed alternatives demonstrated reduced adhesion and more variable failure behavior, underscoring the need for further development in bonding protocols and resin formulation. Although the sample size was adequate for statistical analysis, the study was limited to a restricted number of materials and manufacturers, which does not allow the findings to be generalized to all possible tooth–base resin combinations. Additionally, the use of different bonding systems for milled and printed groups, in line with manufacturer recommendations, may have influenced the results, making it difficult to determine whether the lower SBS in printed groups was due to the resin itself or to the performance of the bonding agent. Future studies should compare standardized bonding protocols across materials to isolate this effect.

## 5. Conclusions

Within the limitations of this study, milled bases appear to represent a reliable option in digital prosthodontics, while printed bases require protocol optimization before long term clinical use. Future investigations should include long-term fatigue tests, thermal cycling, and evaluation of different printing resins and bonding strategies.

## Figures and Tables

**Table 1:** Groups, name of materials, manufacturer and chemical composition.

Group Name	Base Type	Resin Base Manufacturer	Chemical Composition	Tooth Type	Tooth Manufacturer	Chemical Composition
CB-CT	Conventional Base	Probase Hot (Ivoclar Vivadent, Liechtenstein)	PMMA, cross-linked with butanediol dimethacrylate	Conventional Tooth	SPE (Ivoclar Vivadent, Liechtenstein)	PMMA, microfilled composite
MB-CT	Milled Base	Ivobase (Ivoclar Vivadent, Liechtenstein)	Pre-polymerized PMMA	Conventional Tooth (SEP)	SPE (Ivoclar Vivadent, Liechtenstein)	PMMA, microfilled composite
MB-PT	Milled Base	Ivobase (Ivoclar Vivadent, Liechtenstein)	Pre-polymerized PMMA	Printed Tooth	Varseo Smile Teeth (Bego, Germany)	Methacrylate-based light-curing resin
MB-MT	Milled Base	Ivotion base (Ivoclar Vivadent, Liechtenstein)	Multilayered PMMA	Milled Tooth	Ivotion Dent Multi (Ivoclar Vivadent, Liechtenstein)	Multilayered PMMA
PB-CT	Printed Base	V-Print (VOCO, Germany)	Light-curing resin based on dimethacrylates	Conventional Tooth (SEP)	SPE (Ivoclar Vivadent, Liechtenstein)	PMMA, microfilled composite
PB-PT	Printed Base	V-Print (VOCO, Germany)	Light-curing resin based on dimethacrylates	Printed Tooth (Smile Bego)	Varseo Smile Teeth (Bego, Germany)	Methacrylate-based light-curing resin

**Table 2:** Shear Bond Strength (SBS) Values for All Groups.

Group	Mean SBS (MPa)	Standard Deviation
CB-CT	14.9	3.69 A
MB-CT	12.43	2.31 A
MB-PT	12.16	3.14 A
MB-MT	11.71	2.91 B
PB-CT	12.28	2.37 B
PB-PT	8.77	3.52 C
PB-MT	6.58	3.41 C

Different uppercase letters indicate statistically significant differences between groups (*p* < 0.05). CB-CT, conventional base–conventional tooth; MB-CT, milled base–conventional tooth; MB-PT, milled base–printed tooth; MB-MT, milled base–milled tooth; PB-CT, printed base–conventional tooth; PB-PT, printed base–printed tooth; PB-MT, printed base–milled tooth.

**Table 3:** Distribution of failure modes and mean ± SD (MPa) shear bond strength.

Group	Adhesive (MPa)	Cohesive (MPa)	Mixed (MPa)
CB-CT	15.11 ± 5.48 (2) A,a	15.57 ± 3.71(10) A,a	12.55 ± 2.79 (3) A,a
MB-CT	11.53 ± 1.25 (2) A,a	12.92 ± 2.18 (12) B,a	8.41(1) A,a
MB-PT	5.75 (1) C,a	13.44 ± 3.16 (8) A,a	11.51 ± 1.49(6) A,a
MB-MT	–	11.56 ± 3.05 (11) A,b	12.11 ± 2.85(4) A,a
PB-CT	9.76 (1) B,a	12.96 ± 2.38 (11) A,a	10.59 ± 0.94 (3) B,a
PB-MT	3.45 ± 1.12 (3) A,b	9.55 ± 4.24 (5) B,b	5.97 ± 1.34(7) A,b
PB-PT	5.49 ± 1.37 (6) C,b	13.24 ± 1.44 (4) A,a	9.13 ± 1.81 (5) B,a

Note: (n) = number of specimens. Uppercase letters (A–C) indicate differences between failure modes within each group. Lowercase letters (a,b) indicate differences between groups for the same failure mode (*p* < 0.05). “–“ = no specimens recorded.

**Table 4:** Comparing mean shear bond strength of failure mode.

Failure Mode	Mean ± SD (MPa) (n)
Adhesive	7.5 ± 4.4 (15) B
Cohesive	12.9 ± 3.2 (61) A
Mixed	9.7 ± 3.0 (29) B

Different uppercase letters indicate statistically significant differences between failure modes (Bonferroni post hoc, *p* < 0.05).

## Data Availability

The data presented in this study are available on request from the corresponding author. The data are not publicly available due to university policy.
